# Variability of sleep bruxism—findings from consecutive nights of monitoring

**DOI:** 10.1007/s00784-021-04314-8

**Published:** 2021-12-03

**Authors:** Brigitte Ohlmann, Wolfgang Bömicke, Rouven Behnisch, Peter Rammelsberg, Marc Schmitter

**Affiliations:** 1 Department of Prosthodontics, University of Heidelberg, Im Neuenheimer Feld 400, 69120 Heidelberg, Germany; 2 Institute of Medical Biometry and Informatics (IMBI), University of Heidelberg, Im Neuenheimer Feld 130.3, 69120 Heidelberg, Germany; 3 Department of Prosthodontics, University of Würzburg, Pleicherwall 2, 97070 Würzburg, Germany

**Keywords:** Sleep bruxism, Fluctuation, Electromyography, Electrocardiography, Portable

## Abstract

**Objectives:**

To determine sleep bruxism (SB) behavior during five consecutive nights and to identify correlations between SB episodes per hour (SB index) and sleep-time masseter-muscle activity (sMMA).

**Material and methods:**

Thirty-one participants were included in the study. Of these, 10 were classified as sleep bruxers (group SB-1) and nine as non-sleep bruxers (group non-SB). The bruxism status of these 19 patients was identified by means of questionnaires, an assessment of clinical symptoms, and electromyographic/electrocardiographic data (Bruxoff® device). The remaining 12 participants were also identified as bruxers, but based exclusively on data from the Bruxoff device (group SB-2). Data analysis included descriptive statistics and Spearman’s correlation to assess the relationship between the SB index and sMMA.

**Results:**

Participants in group SB-1 showed an overall mean SB index of 3.1 ± 1.6 and a mean total sMMA per night of 62.9 ± 38.3. Participants in group SB-2 had an overall mean SB index of 2.7 ± 1.5 and a mean total sMMA of 56.0 ± 29.3. In the non-SB group, participants showed an overall mean SB index of 0.8 ± 0.5 and a mean total sMMA of 56.8 ± 30.3. Spearman’s correlation yielded values of − 0.27 to 0.71 for the correlation between sMMA and SB index.

**Conclusions:**

The data revealed variable SB activity and the absence of a reliable correlation between sMMA and the SB index.

**Clinical relevance:**

The high variation in SB activity and lack of correlation between sMMA and the SB index should be considered when diagnosing SB.

**Trial registration:**

Clinical Trials [NIH], clinical trial no. NCT03039985.

## Introduction

Bruxism can have detrimental effects such as abnormal tooth wear [1], failed dental restorations [2], masticatory muscle pain [3], and headaches [4]. Consequently, reliable diagnosis of bruxism is of great importance to many different medical disciplines. The consequences of bruxism adversely affect a patient’s quality of life [5, 6] and entail dental follow-up costs [7]. How sleep bruxism (SB) is defined and classified has varied in recent decades. These changes reflect the high degree of activity in this field of research, which aims to clarify the symptoms, causes, and consequences of bruxism.

In 2013, a group of bruxism experts reached international consensus on a definition for SB, namely, repetitive jaw-muscle activity characterized by clenching or grinding of the teeth and/or bracing or thrusting of the mandible with circadian manifestations [8, 9]. A diagnostic grading system was also proposed [8], which suggested the following three bruxism categories: (i) possible SB (based on self-reporting only), (ii) probable SB (based on self-reporting and clinical signs), and (iii) definite SB (based on self-reporting, clinical signs, and polysomnography). This SB grading system was revised in 2018 [10], and it was decided that only a positive instrumental assessment (with or without positive self-reporting and/or positive clinical signs) should be used to diagnose definite SB. Furthermore, it was recommended that the focus of SB diagnosis might need to shift from the concept of SB events to the scoring of masticatory muscle activity (MMA) [11].

However, the experts also firmly agreed that more research is required to determine how to assess SB in an ideal manner. To ensure that a sample of real-life SB activity is representative, it is essential to know the variability of the collected data and the correlation between SB events and MMA.

Studies of SB activity over more than one recorded night have yielded heterogenous results [12–16]. Whereas some studies concluded that the SB diagnosis remains quite constant over time [14] or that sleep-time masticatory muscle activity (sMMA) shows no significant differences over a prolonged recording span [17], other studies found high variation in SB activity [12, 13, 16].

Starting from this point, it is therefore desirable to collect data from individuals over extended periods, preferably in the form of polysomnography measurements. However, polysomnography is very expensive and requires complex technical equipment [18]. Moreover, polysomnography provides information on the patient’s oral behavior in an unusual situation (i.e., in hospital) and not in a familiar domestic environment, as would be desirable [19].

Following the introduction of the portable Bruxoff® device, which can diagnose SB with high sensitivity and specificity by combining both electromyographic and electrocardiographic data [19], it appears that reliable data can be obtained to diagnose SB under domestic conditions. The objectives of this study are therefore as follows:

1. to determine and compare variation in SB activity—recorded by the Bruxoff device over five consecutive nights—among different groups of individuals with SB.
2. to identify correlations between the number of SB episodes per hour (SB index; recorded using the Bruxoff device) and sMMA.

We hypothesized that the different SB groups would not differ regarding variation in SB activity. With regard to possible correlations between SB episodes per hour and sMMA, we hypothesized that there would be a consistent positive correlation between SB index and sMMA.

## Material and methods

### Study design and approval

This study was approved by the local Ethics Committee, protocol no. S-312/2014. All participants received information about the procedure and the possible risks and benefits of the study, and all participants gave informed consent.

### Participants

The individuals evaluated in this study were taken from a randomized clinical study evaluating single ceramic molar crowns in patients with and without SB (clinical trial no. NCT03039985). Thus, all patients included in the study required a molar crown with a natural antagonist. Patients who were younger than 18 or not permitted to take out a contract were excluded from the study, as were patients who were pregnant or lactating, had acute neuropsychiatric diseases, hemorrhagic diathesis, or a heart pacemaker, or who had a known allergic reaction to the materials used.

### Self-reported bruxism

Two questionnaires were used for self-reporting of bruxism:

#### The first questionnaire, by Paesani et al. [20], contains the following items for assessing clenching/grinding while awake and asleep:

- Are you aware of the fact that you grind your teeth during sleep?
- Did anyone tell you that you grind your teeth during sleep?
- On morning awakening or on awakenings during the night, do you have your jaws thrust or braced?
- Do you grind/clench your teeth while awake?

According to Paesani et al., bruxism is present if one of the items is answered “yes.”

#### The second questionnaire, by Raphael et al. [21], is a structured interview that contains the following items for assessing sleep clenching/grinding:

- Have you ever been told you grind your teeth at night during sleep?
- Have you ever noticed that you grind your teeth at night during sleep?
- In the last two weeks, have you been told you grind your teeth at night during sleep?
- In the last two weeks, have you noticed that you grind your teeth at night during sleep?

According to Raphael et al., bruxism is present if one of the items is answered “yes.”

In this study, self-reported bruxism was recorded as either “yes” or “no” if both questionnaires produced identical results.

### Clinical signs of bruxism

A clinical examination was conducted to assess the presence of four clinical signs of bruxism, with each item answered “yes” or “no”:

- Abnormal tooth wear
- Impressions of teeth in the buccal region
- Impressions of teeth on the tongue
- Masseter-muscle hypertrophy

In this study, clinical signs of bruxism were present if one of the four items was answered “yes.”

The clinical examination was performed by a single examiner, who was unaware of the results of the questionnaires and Bruxoff recordings. Additionally, all participants were assessed using the complete Research Diagnostic Criteria for Temporomandibular Disorders (RDC/TMD) [22] diagnostic protocol.

### Instrumental diagnosis of SB

To make the diagnosis of bruxism, all participants used a portable electromyography and electrocardiography device (Bruxoff) for five consecutive nights. The Bruxoff device is a type 3 cardio electromyographic polygraphy recorder that uses three channels: two channels are used to acquire electromyographic data bilaterally from the masseter muscle, and the third channel is used to acquire heart frequency. Participants received a detailed explanation and a written step-by-step description of how to use the Bruxoff device. They were instructed to perform three maximum voluntary clenchings (MCVs) at the start of each night and examination. The software (Bruxmeter®) automatically scores the total number of sMMA events as well as the tonic, phasic, and mixed sMMA. It also records the total number of SB episodes per night (SB/n) and the number of SB episodes per hour (SB index). The software classifies a potential event as SB if the surface electromyographic burst (bilaterally from the masseter) is greater than 10% of MVC and if it immediately follows (1–5 s interval) an increase in heart rate of 20% with respect to the baseline [23].

Measurement data were considered inadequate and excluded from further analysis if any of the following happened during recording:

- Disconnection of at least one of the electrodes (masseter and thorax)
- Unsuccessful calibration of one or both masseters
- More than 100 saturation signals
- More than 100 data interferences

Participants were included in the study if measurements were successfully recorded during at least four out of five consecutive nights and for at least four hours per night.

In this study, a diagnosis of (moderate) SB was made if more than two SB episodes per hour of sleep (SB index > 2) [18] were determined for one or more nights.

### Study groups

For further analysis, participants were divided into three groups:

-
  -
    - Group SB-1: positive instrumental diagnosis plus positive self-reported bruxism plus positive clinical signs of bruxism.
-
  -
    - Group SB-2: positive instrumental diagnosis, regardless of self-reporting and/or clinical signs of bruxism.
-
  -
    - Group non-SB: negative self-reported bruxism plus negative clinical signs of bruxism plus negative instrumental diagnosis.

### Statistical analysis

Data were evaluated by use of statistical software (SPSS 24; IBM Corp., New York, United States, and SAS version 9.4; SAS Institute Inc., Cary, United States) with the participant as the statistical unit. Data were presented for each night for each participant, and the coefficient of variation for the SB index was calculated for each group of patients. Statistical evaluation included Spearman’s correlation and analysis of variance (ANOVA).

## Results

The study population of 31 participants comprised 10 men (mean age 53.9 ± 15.1 years) and 21 women (mean age 50.8 ± 11.6 years). Regarding the RDC/TMD assessment, a total of eight participants had an RDC/TMD diagnosis: two participants were diagnosed with myofascial pain (both in group SB-1), one with arthralgia (in group SB-1), and five with disc displacement with reduction (one in group SB-1, one in group SB-2, and three in group non-SB).

### Group SB-1 (N = 10)

The following results were recorded for participants in group SB-1: an overall mean SB index of 3.1 ± 1.6, a mean total number of SB episodes per night (SB/n) of 19.8 ± 10.1, and a mean sMMA per night of 62.9 ± 38.3 masseter contractions. The lowest mean SB index per participant was 1.5 ± 0.56, and the highest was 5.8 ± 2.13. An SB index of > 2 during all recorded nights was recorded for six out of the 10 participants, and the remaining four participants had an SB index of ≤ 2 during 1–3 recorded nights. The coefficient of variation is presented in Table 1. Table 2 provides detailed information on the number of bruxism episodes per hour (SB index) and per night (SB/n) and the total number of sMMA events per night for the participants in group SB-1.

**Table 1:** Coefficient of variation (CV) of bruxism index in the three groups

CV	Group SB-1	Group SB-2	Group non-SB	Total	*p* value†
N	10	12	9	31	0.301
Mean	0.41	0.36	0.57	0.44	
SD	0.24	0.23	0.43	0.31	
Median	0.36	0.26	0.53	0.35	
Q1–Q3	0.29–0.53	0.2–0.55	0.3–0.69	0.24–0.56	
Min–max	0.12–0.98	0.089–0.77	0.15–1.5	0.089–1.5	

^†^*F* test (ANOVA)

**Table 2:** Bruxism episodes per hour (SB index) and per night (SB/N) and sleep-time masseter-muscle activity (sMMA) for participants in group SB-1

P no^†^	Night 1			Night 2			Night 3			Night 4			Night 5		
	SB index	SB/n	sMMA	SB index	SB/n	sMMA	SB index	SB/n	sMMA	SB index	SB/n	sMMA	SB index	SB/n	sMMA
1	IM^‡^	IM	IM	3.3	25	111	0.7	5	9	3.6	23	110	4.4	27	58
2	4.4	27	205	2.5	16	182	3.8	24	90	2.5	17	78	IM	IM	IM
3	3.2	22	50	2.1	15	85	2.0	12	57	1.5	10	59	2.6	17	47
4	IM	IM	IM	3.7	18	50	2.7	22	60	2.7	21	69	2.4	16	40
5	6.3	32	37	IM	IM	IM	3.3	18	27	8.4	30	42	5.2	25	30
6	3.6	27	37	4.7	42	66	3.6	29	52	7.7	58	114	4.5	30	53
7	2.5	17	60	2.5	20	101	2.2	14	92	2.1	11	36	IM	IM	IM
8	1.6	6	24	1.1	5	26	2.3	14	89	3.9	13	40	2.3	9	47
9	IM	IM	IM	1.0	6	23	2.2	15	68	1.1	8	37	1.7	16	72
10	2.1	17	34	3.6	24	56	3.0	16	35	3.5	22	50	4.5	32	60

^†^*P* no participant number, ^‡^*IM* incorrect measurement

### Group SB-2 (N = 12)

One participant showed positive clinical signs of bruxism without a positive self-report, and one participant had a positive self-report without clinical signs of bruxism. The remaining 10 participants had neither positive clinical signs nor a positive self-report.

For participants in the SB-2 group, an overall mean SB index of 2.7 ± 1.5, a mean total SB/n of 17.1 ± 9.7, and a mean sMMA per night of 56.0 ± 29.3 were observed. The lowest mean SB index per participant was 1.3 ± 0.89, and the highest was 5 ± 1.87. An SB index of > 2 was recorded for six out of 12 participants during all recorded nights, and the remaining seven participants had an SB index of ≤ 2 during 1–3 recorded nights. The coefficient of variation is presented in Table 1. Table 3 provides detailed information on the SB index, SB/n, and total number of sMMA events per night for the participants in group SB-2.

**Table 3:** Bruxism episodes per hour (SB index) and per night (SB/n) and sleep-time masseter-muscle activity (sMMA) for participants in group SB-2

P no^†^	Night 1			Night 2			Night 3			Night 4			Night 5		
	SB index	SB/n	sMMA	SB index	SB/n	sMMA	SB index	SB/n	sMMA	SB index	SB/n	sMMA	SB index	SB/n	sMMA
1	1.1	7	56	3.1	17	60	2.3	14	30	4.4	27	78	1.3	8	23
2	3.6	14	21	IM^‡^	IM	IM	2.9	10	20	5.1	19	40	5.7	26	49
3	1.5	9	45	0.2	1	52	2.4	12	47	0.6	4	32	1.8	13	50
4	2.1	16	59	3.3	25	55	3.1	26	69	IM	IM	IM	3.4	23	58
5	1.8	12	63	2.8	22	109	0.5	3	72	0.6	4	102	IM	IM	IM
6	2.0	15	68	2.5	20	43	IM	IM	IM	2.4	21	69	2.1	6	16
7	0.3	2	9	0.7	6	10	5.4	34	79	IM	IM	IM	3.3	32	47
8	1.5	12	29	2.3	19	41	1.9	13	27	2.9	22	45	0.0	0	0
9	3.5	17	141	IM	IM	IM	3.6	21	79	4.3	24	117	3.3	24	57
10	2.9	16	64	2.7	18	110	3.7	23	74	2.1	13	76	2.1	12	38
11	7.1	55	72	6.0	29	48	3.8	21	28	3.1	15	25	IM	IM	IM
12	1.7	15	75	2.1	18	110	2.8	25	68	3.1	27	70	2.2	18	45

^†^*P* no participant number, ^‡^*IM* incorrect measurement

### Group non-SB (N = 9)

Participants in the non-SB group had an overall mean SB index of 0.8 ± 0.5, a mean total SB/n of 5.7 ± 3.4, and a mean total sMMA per night of 56.8 ± 30.3. The lowest mean SB index per participant was 0.12 ± 0.19, and the highest was 1.32 ± 0.21. The coefficient of variation is presented in Table 1. Table 4 contains detailed information on the SB index, SB/n, and total number of sMMA events per night for participants in the non-SB group.

**Table 4:** Bruxism episodes per hour (SB index) and per night (SB/n) and sleep-time masseter-muscle activity (sMMA) for participants in group non-SB

P no^†^	Night 1			Night 2			Night 3			Night 4			Night 5		
	SB index	SB/n	sMMA	SB index	SB/n	sMMA	SB index	SB/n	sMMA	SB index	SB/n	sMMA	SB index	SB/n	sMMA
1	0.8	6	61	0.7	5	36	0.7	5	36	1.0	8	42	0.8	3	33
2	1.9	12	60	1.7	12	94	0.9	5	44	0.5	3	79	IM	IM	IM
3	1.1	9	95	0.5	4	121	1.0	8	82	0.7	6	90	IM	IM	IM
4	1.1	10	62	0.5	4	89	0.1	1	82	1.0	6	63	IM	IM	IM
5	IM^‡^	IM	IM	0.2	3	18	1.1	8	84	1.3	9	143	1.1	6	98
6	IM	IM	IM	1.6	12	35	1.1	7	33	1.3	10	26	1.3	9	52
7	0.2	1	31	1.6	9	37	1.0	6	47	0.8	4	18	0.0	0	15
8	IM	IM	IM	0.4	3	53	0.0	0	58	0.0	0	43	0.1	1	74
9	1.1	6	32	IM	IM	IM	0.9	7	34	0.9	5	39	0.5	3	19

^†^*P* no participant number, ^‡^*IM* incorrect measurement

### Correlation between SB index and masseter contractions

For all groups, Spearman’s correlation did not confirm a consistent correlation between the SB index and masseter contractions, including phasic, tonic, and mixed contractions. Detailed correlations are presented in Table 5.

**Table 5:** Correlations between sleep bruxism index and sleep-time masseter-muscle activity (sMMA; total, phasic, tonic, and mixed contractions)

Group	Night	Total sMMA	Phasic sMMA	Tonic sMMA	Mixed sMMA
SB-1	1	0.54	0.68	0.38	− 0.02
SB-1	2	0.25	0.37	0.08	0.28
SB-1	3	0.09	− 0.01	0.02	0.13
SB-1	4	0.35	0.05	0.41	0.25
SB-1	5	− 0.27	− 0.27	0.01	− 0.06
SB-2	1	0.49	0.14	0.20	0.25
SB-2	2	0.23	− 0.19	− 0.24	− 0.22
SB-2	3	0.40	− 0.27	− 0.23	− 0.43
SB-2	4	0.03	− 0.26	− 0.06	0.12
SB-2	5	0.71	0.70	0.49	0.45
Non-SB	1	0.33	0.46	0.71	− 0.48
Non-SB	2	0.19	0.47	0.63	− 0.16
Non-SB	3	− 0.03	− 0.18	0.04	− 0.38
Non-SB	4	− 0.08	0.08	0.03	− 0.34
Non-SB	5	0.54	− 0.31	− 0.09	n.a

## Discussion

This study aimed to determine SB activity over five consecutive nights and to compare SB activity among three different SB groups. A further aim was to identify correlations between the SB index of the Bruxoff device and sMMA. Based on the results, the first hypothesis can be accepted: The three SB groups did not differ regarding variation in SB activity. However, the second hypothesis of a consistent positive correlation between sMMA and SB index must be rejected.

Because SB has potentially detrimental consequences, reliable diagnosis of this condition is of great interest to both general practitioners and dentists. Following the 2018 revision of the previous international consensus on bruxism [24], a positive instrumental assessment is considered the most important diagnostic criterion for distinguishing between sleep bruxers and non-bruxers. Moreover, it was recommended that the focus of SB diagnosis might need to shift from the concept of SB events to the scoring of MMA [11].

Although the data in the literature are inconsistent, it is suspected that SB varies greatly over time [12–14, 16]. Some studies have reported variation in repeated measurements, with subsequent measurements more than four times higher than the values obtained during the first round of measurements [16]. Other studies, in contrast, found moderate variation in the number of SB events per hour [13] or a stable diagnosis of SB over time [14]. In the present study, high variability was found for the SB index. With regard to the previously determined cut-off value of > 2 SB episodes/h [25] for moderate SB, only six out of 10 sleep bruxers with clinical signs and self-reported bruxism received a diagnosis of SB from the Bruxoff during all nights. Excluding the first night, no differences could be proved regarding a false-positive or false-negative diagnosis. Comparable results were seen if the cut-off value was set at > 4 episodes/h. The SB diagnosis for the SB-2 group, whose participants had no self-reported bruxism and/or clinical signs of bruxism, was also not stable over the recorded nights. These results are consistent with those of Muzalev et al. [16], who found that the number of bruxism episodes/h varied considerably for two patients examined over six weeks. Miettinen et al. presented similar results [12], also finding substantial inter-night variability over three consecutive nights, as did Van der Zaag et al. [13]. Van der Zaag et al. investigated the nature of SB fluctuations by studying the outcome variables of polysomnography recordings. They found variation of ± 2.77 bruxism episodes for bruxers and ± 1.11 for non-bruxers, which is comparable to the results of this study.

As in the current study, these previous studies used a cut-off point of bruxism events to distinguish bruxers from non-bruxers. Some authors have recommended using cut-off bands instead of cut-off points [13], or to generally abolish cut-off points, with a focus instead of bruxism-related muscle activity [24]. It was suggested that the duration and amount of muscle activity are better predictors of an SB diagnosis than the number of events is, and should therefore be used instead of diagnosis cut-off points. In contrast, polysomnography studies point out the risk of overestimation in the absence of an audio–video recording [26] or show that relying exclusively on electromyography data is unreliable [27]. The present study found no consistent correlation between masseter contractions and the SB index. This suggests that although the previously mentioned approach [24] might suffice for some patients, it is not suitable for all. A recent study investigated the frequency of SB behaviors (SB index and sMMA) over a four-night recording, again using the Bruxoff device. The SB index results from that study are comparable to those in the present study, but the authors concluded that differences over the recording span were not significant [17]. Our study presents similar data; nevertheless, the data are difficult to compare due to differences in patient selection. Whereas the study by Colonna et al. [17] concentrated on healthy young individuals, the participants in our study were older, and those who had temporomandibular disorders were not excluded from taking part. Moreover, the patients were classified according to their bruxism-related clinical findings and self-reports in order to detect possible variations among the groups. Furthermore, the study by Colonna et al. [17] concentrated on tonic, phasic, and mixed masseter contractions per hour of sleep, whereas our study concentrated on the total masseter contractions per night and presented data for each participant and night. Apart from these differences, the average SB index over the recording nights in the study by Colonna et al. is comparable to the results of the present study.

Our study does, however, have some limitations. First, the patients were pre-selected from a clinical study of dental restorations. Furthermore, the small sample size means the risk of error is higher than it would be for a larger sample. The use of the Bruxoff device might constitute another weakness. Although this portable and simple screening device has high sensitivity for detecting SB [19], it cannot be considered a reliable replacement for type 1 polysomnography, because it does not have audio–video recording or electroencephalogram monitoring [28]. Consequently, it cannot register visual or auditive activity, and/or it remains unclear if there are times of “wake after sleep onset” during recording.

Future research should investigate the instrumental assessment of bruxism over prolonged periods [24], particularly with larger sample sizes.

## Conclusions

Our study results indicate that the number of SB episodes and sMMA events varies greatly over time. Furthermore, the data revealed the absence of a reliable correlation between masseter contractions and the SB index. This finding indicates that the recently revised criteria for diagnosing SB should be critically reexamined. Further studies on this topic are warranted.

## References

[1] Yoshida Y, Suganuma T, Takaba M, Ono Y, Abe Y, Yoshizawa S, Sakai T, Yoshizawa A, Nakamura H, Kawana F, Baba K (2017). Association between patterns of jaw motor activity during sleep and clinical signs and symptoms of sleep bruxism. J Sleep Res.

[2] Johansson A, Omar R, Carlsson GE (2011). Bruxism and prosthetic treatment: a critical review. J Prosthodont Res.

[3] Johansson A, Unell L, Carlsson GE, Soderfeldt B, Halling A (2003). Gender difference in symptoms related to temporomandibular disorders in a population of 50-year-old subjects. J Orofac Pain.

[4] Vieira KRM, Folchini CM, Heyde M, Stuginski-Barbosa J, Kowacs PA, Piovesan EJ (2020). Wake-up headache is associated with sleep bruxism. Headache.

[5] Yıldırım G, Erol F, Güven MC, Şakar O (2020). Evaluation of the effects of bruxism on oral health-related quality of life in adults. Cranio.

[6] Carvalho Ade M, Lima Mde D, Silva JM, Neta NB, Moura Lde F (2015). Bruxism and quality of life in schoolchildren aged 11 to 14. Cien Saude Colet.

[7] De Boever AL, Keersmaekers K, Vanmaele G, Kerschbaum T, Theuniers G, De Boever JA (2006). Prosthetic complications in fixed endosseous implant-borne reconstructions after an observations period of at least 40 months. J Oral Rehabil.

[8] Lobbezoo F, Ahlberg J, Glaros AG, Kato T, Koyano K, Lavigne GJ, de Leeuw R, Manfredini D, Svensson P, Winocur E (2013). Bruxism defined and graded: an international consensus. J Oral Rehabil.

[9] Manfredini D, Lobbezoo F (2009). Role of psychosocial factors in the etiology of bruxism. J Orofac Pain.

[10] Lobbezoo F, Visscher CM, Koutris M (2018). Bruxism in dentists' families. J Oral Rehabil.

[11] Thymi M, Lobbezoo F, Aarab G, Ahlberg J, Baba K, Carra MC, Gallo LM, De Laat A, Manfredini D, Lavigne G, Svensson P (2021). Signal acquisition and analysis of ambulatory electromyographic recordings for the assessment of sleep bruxism: a scoping review. J Oral Rehabil.

[12] Miettinen T, Myllymaa K, Hukkanen T, Töyräs J, Sipilä K, Myllymaa S (2018). Home polysomnography reveals a first-night effect in patients with low sleep bruxism activity. J Clin Sleep Med.

[13] Van Der Zaag J, Lobbezoo F, Visscher CM, Hamburger HL, Naeije M (2008). Time-variant nature of sleep bruxism outcome variables using ambulatory polysomnography: implications for recognition and therapy evaluation. J Oral Rehabil.

[14] Lavigne GJ, Guitard F, Rompre PH, Montplaisir JY (2001). Variability in sleep bruxism activity over time. J Sleep Res.

[15] Restrepo C, Lobbezoo F, Castrillon E, Svensson P, Santamaria A, Alvarez C, Manrique R, Manfredini D (2018). Agreement between jaw-muscle activity measurement with portable single-channel electromyography and polysomnography in children. Int J Paediatr Dent.

[16] Muzalev K, Visscher CM, Koutris M (2018). Long-term variability of sleep bruxism and psychological stress in patients with jaw-muscle pain: report of two longitudinal clinical cases. J Oral Rehabil.

[17] Colonna A, Segù M, Lombardo L, Manfredini D (2021) Frequency of sleep bruxism behaviors in healthy young adults over a four-night recording span in the home environment. Appl Sci 11:195. 10.3390/app11010195

[18] Manfredini D, Ahlberg J, Castroflorio T, Poggio CE, Guarda-Nardini L, Lobbezoo F (2014). Diagnostic accuracy of portable instrumental devices to measure sleep bruxism: a systematic literature review of polysomnographic studies. J Oral Rehabil.

[19] Castroflorio T, Deregibus A, Bargellini A, Debernardi C, Manfredini D (2014). Detection of sleep bruxism: comparison between an electromyographic and electrocardiographic portable holter and polysomnography. J Oral Rehabil.

[20] Paesani DA, Lobbezoo F, Gelos C, Guarda-Nardini L, Ahlberg J, Manfredini D (2013). Correlation between self-reported and clinically based diagnoses of bruxism in temporomandibular disorders patients. J Oral Rehabil.

[21] Raphael KG, Sirois DA, Janal MN, Wigren PE, Dubrovsky B, Nemelivsky LV, Klausner JJ, Krieger AC, Lavigne GJ (2012). Sleep bruxism and myofascial temporomandibular disorders: a laboratory-based polysomnographic investigation. J Am Dent Assoc.

[22] Dworkin SF, LeResche L (1992). Research diagnostic criteria for temporomandibular disorders: review, criteria, examinations and specifications, critique. J Craniomandib Disord.

[23] Deregibus A, Castroflorio T, Bargellini A, Debernardi C (2014). Reliability of a portable device for the detection of sleep bruxism. Clin Oral Investig.

[24] Lobbezoo F, Ahlberg J (2018). International consensus on the assessment of bruxism: Report of a work in progress. J Oral Rehabil.

[25] Rompre PH, Daigle-Landry D, Guitard F, Montplaisir JY, Lavigne GJ (2007). Identification of a sleep bruxism subgroup with a higher risk of pain. J Dent Res.

[26] Carra MC, Huynh N, Lavigne GJ (2015). Diagnostic accuracy of sleep bruxism scoring in absence of audio-video recording: a pilot study. Sleep Breath.

[27] Miettinen T, Myllymaa K, Muraja-Murro A, Westeren-Punnonen S, Hukkanen T, Töyräs J, Lappalainen R, Mervaala E, Sipilä K, Myllymaa S (2020). Polysomnographic scoring of sleep bruxism events is accurate even in the absence of video recording but unreliable with EMG-only setups. Sleep Breath.

[28] Saczuk K, Lapinska B, Wilmont P, Pawlak L, Lukomska-Szymanska M (2019). The Bruxoff device as a screening method for sleep bruxism in dental practice. J Clin Med.

